# Decoding the interactome for cyclic-di-AMP-producing enzyme diadenylate cyclase

**DOI:** 10.1128/msystems.01727-25

**Published:** 2026-06-03

**Authors:** Rong Mu, Baotong Xie, Stephanie Momeni, Hui Wu

**Affiliations:** 1 Department of Biomaterial and Biomedical Sciences, School of Dentistry, Oregon Health and Science University, Portland, Oregon, USA; Northwestern University Feinberg School of Medicine, Chicago, Illinois, USA

**Keywords:** diadenylate cyclase, *Streptococcus mutans*, protein-protein interactions

## Abstract

In many gram-positive bacteria, the diadenylate cyclase DacA synthesizes the essential second messenger c-di-AMP. While regulators like GlmM and CdaR are known, the broader DacA interactome and its functional relevance remain poorly defined. Using *Streptococcus mutans* as a model, we identified novel protein-protein interactions using co-immunoprecipitation and mass spectrometry approaches. We identified 22 candidate interactors under non-crosslinked conditions and 18 under crosslinked conditions, with six shared candidates. Using AlphaFold-guided modeling and experimental split-luciferase complementation, we validated SMU_723—a putative calcium transporter—as a primary binding partner. Targeted mutagenesis identified threonine 147, glutamine 148, and threonine 149 as the key interacting interface; mutations in these residues impaired binding, confirming their structural importance. Disruption of the DacA–SMU_723 interaction resulted in a significant reduction in intracellular c-di-AMP levels, supporting a role for SMU_723 in modulating DacA enzymatic activity. Deletion of SMU_723 recapitulated several phenotypes observed in the ∆*dacA* strain, including an extended lag phase, altered cell morphology, reduced acid production and acid tolerance, impaired sorbitol metabolism, decreased colonization in the *Drosophila* model, and delayed growth following calcium stimulation. However, ∆SMU_723 phenotypes were generally less severe than those of ∆*dacA*, indicating partial functional overlap rather than a complete phenocopy. Together, this study identifies a functional link between SMU_723 and DacA that modulates c-di-AMP levels and calcium response and is vital for the survival of *S. mutans*.

**IMPORTANCE:**

Mapping the DacA interactome reveals how environmental and intracellular cues tune c-di-AMP signaling to control stress adaptation, ion balance, and virulence traits in *Streptococcus mutans*. By identifying new DacA-associated proteins and validating SMU_723 as a previously unrecognized interactor with genetic and phenotypic linkage to DacA, this study provides new insights into the mechanistic framework for c-di-AMP regulation in *S. mutans*. The connection between DacA and a putative calcium transporter highlights a plausible axis that couples second-messenger signaling to calcium homeostasis, with implications for biofilm physiology and pathogenesis. These insights open new avenues to therapeutically modulate c-di-AMP pathways.

## INTRODUCTION

Cyclic dimeric adenosine monophosphate (c-di-AMP) (1) is a pivotal bacterial second messenger that regulates core physiological processes, including potassium homeostasis, osmotic stress responses, cell wall integrity, and virulence (2–4). Its synthesis is catalyzed by diadenylate cyclases (DACs), which convert two ATP molecules into c-di-AMP. Across bacteria and some archaea, DACs comprise multiple families distinguished by domain architecture and phylogeny. The CdaA family (hereafter) is among the most widespread and best characterized: it typically contains an N-terminal transmembrane (TM) domain (Pfam PF19293) that anchors the enzyme in the cytoplasmic membrane and a conserved C-terminal catalytic domain (Pfam PF02457) that converts ATP molecules into c-di-AMP (5).

CdaA is the sole diadenylate cyclase in streptococci, making it a non-redundant enzyme for c-di-AMP production. Genetic and physiological studies in pathogenic streptococci underscore their centrality. While specific phenotypic contributions are species-dependent, disrupting c-di-AMP homeostasis consistently impairs growth, exacerbates susceptibility to environmental and antibiotic stressors, and attenuates virulence. These impacts are observed across a diverse clinical spectrum by different pathogens, including *Streptococcus pneumoniae* (pneumonia) (6, 7), group A *Streptococcus* (pharyngitis and rheumatic heart disease) (8, 9), *Streptococcus agalactiae* (neonatal sepsis) (10, 11), *Streptococcus mutans* (dental caries) (12–14), *Streptococcus suis* (zoonosis) (15), and *Streptococcus gallolyticus* subsp*. gallolyticus* (endocarditis and colorectal cancer) (16, 17). Collectively, these findings highlight the conserved yet species-specific roles of this second messenger in bacterial pathogenesis.

Although DAC’s enzymatic function is defined, how its activity is regulated in cells remains incompletely understood. Work in *Listeria monocytogenes* and *Bacillus subtilis* established that DacA engages protein-protein interactions with partners encoded in the same operon—most notably CdaR and GlmM—to tune its enzymatic activity (18, 19). In *Lactococcus lactis*, bacterial two-hybrid assays confirm that CdaA physically interacts with GlmM, although the precise mechanism by which GlmM regulates DacA activity remains unclear (20). By contrast, the DacA interactome in streptococci, including *Streptococcus mutans*, has not been characterized, and potential protein partners remain unknown. Given that c-di-AMP is essential in some streptococcal species but dispensable in others—reflecting differences in metabolism and environmental niches (11, 21, 22)—mapping the DacA interaction network is likely to reveal species-specific regulatory logic, potential crosstalk with other signaling and metabolic pathways, and new nodes for therapeutic intervention (11, 21, 22).

Here, we identified DacA-associated proteins in *S. mutans* using combined affinity purification-mass spectrometry (AP-MS) and an *in vivo* split-luciferase complementation assay. We generated a DacA-FLAG strain and identified multiple candidate interactors under both non-crosslinked and crosslinked conditions. SMU_723 was identified as a previously unrecognized DacA binding partner. Structural modeling suggested a direct DacA–SMU_723 interface mediated by residues Thr147, Gln148, and Thr149 in DacA’s catalytic domain; site-directed mutagenesis confirmed their importance for binding. Deletion of SMU_723 phenocopied multiple features of a Δ*dacA* strain, such as prolonged lag phase, aberrant cell morphology, reduced acid production and acid tolerance, impaired sorbitol utilization, diminished colonization in a *Drosophila* model, and delayed growth upon calcium stimulation, pointing to a functional linkage. SMU_723 is a putative calcium transporter, suggesting that DacA may interface with calcium homeostasis or calcium-responsive signaling. Together, these findings expand the DacA regulatory landscape in *S. mutans* and implicate calcium transport as a previously underappreciated dimension of c-di-AMP control.

## RESULTS

### Creating an affinity purification system in *S. mutans*

**Fig 1:**
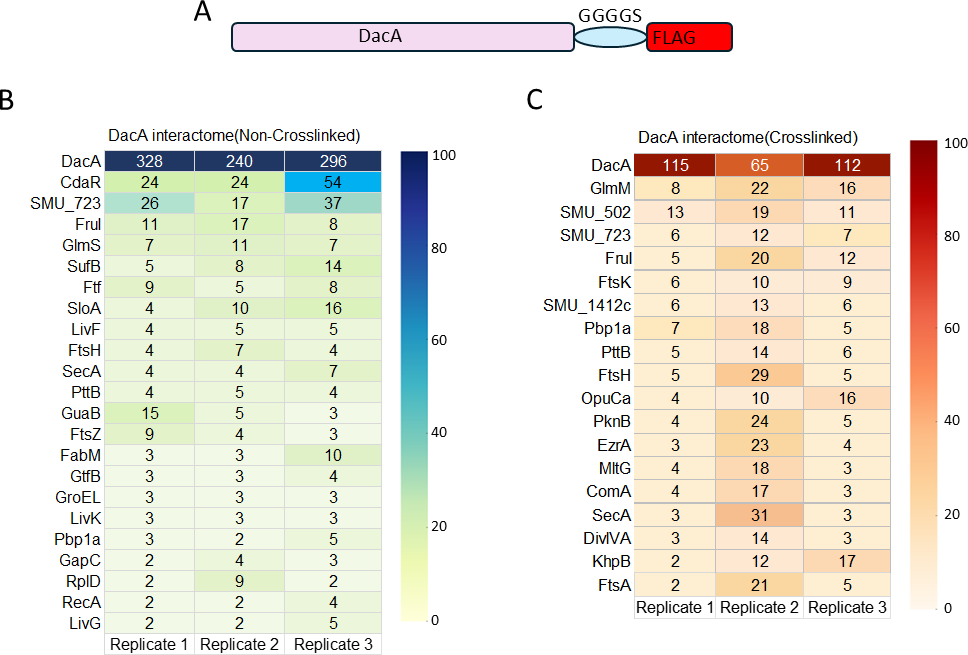
Engineering FLAG-tagged DacA and proteomic analysis of DacA interactome under non-crosslinking and crosslinking conditions. (**A**) Schematic representation of the FLAG-tagged DacA construct. A FLAG epitope tag was fused to the C-terminus of DacA via a flexible GGGGS linker to facilitate co-immunoprecipitation. (**B**) Heat map showing fold changes of proteins co-immunoprecipitated with FLAG-DacA under non-crosslinking conditions. Data represent results from three independent biological replicates. (**C**) Heat map showing fold changes of proteins co-immunoprecipitated with FLAG-DacA under crosslinking conditions. Data represent results from three independent biological replicates. Only proteins enriched ≥2-fold and with spectral counts >10 in the experimental samples were considered for analysis.

To identify potential protein-protein interactions involving DacA, we constructed a *dacA*-FLAG fusion strain in *Streptococcus mutans*, in which a FLAG epitope tag was fused to the C-terminus of DacA via a flexible linker to preserve structural integrity (Fig. 1A). Both non-crosslinked and crosslinked co-immunoprecipitation experiments were performed using mid-log phase cultures of the wild-type *S. mutans* UA159 strain and the engineered DacA-FLAG strain. DacA-containing protein complexes were isolated from cell lysates using anti-FLAG affinity resin, and the immunoprecipitated complexes were subject to mass spectrometry analysis. Specific enrichment of potential interactor peptides was assessed based on spectral count values, representing protein abundance. Proteins identified in the DacA-FLAG strain were compared to those from the wild-type strain lacking the FLAG tag. In total, 22 proteins from the non-crosslinked condition (Fig. 1B) and 18 proteins from the crosslinked condition (Fig. 1C) showed at least a twofold enrichment and had spectral counts greater than 10 in the experimental samples. Six proteins were identified in both conditions. Notably, known DacA regulators within the same genetic locus, such as GlmM and CdaR, were also detected with strong enrichment in the crosslinked and non-crosslinked samples, respectively. Adjacent to these regulators, SMU_723 emerged as a highly enriched protein that was consistently co-purified with DacA under both conditions and showed strong enrichment relative to the no-FLAG control, suggesting it is a specific interaction partner of DacA.

### Validation of protein-protein interaction using a split luciferase complementation assay (SLCA)

**Fig 2:**
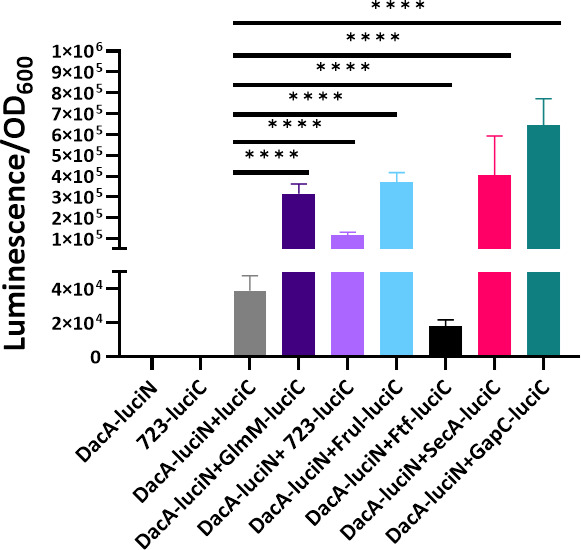
*In vivo* validation of DacA interactors using split luciferase complementation. The N-terminal domain of RenG luciferase (amino acids 1–155) was fused to the C-terminus of DacA, while the C-terminal domain (amino acids 156–314) was fused to the C-terminus of candidate DacA-interacting proteins to generate test strains: DacA-luciN + 723-luciC, DacA-luciN + FruI-luciC, DacA-luciN + Ftf-luciC, DacA-luciN + SecA-luciC, DacA-luciN + GapC-luciC, and DacA-luciN + GlmM-luciC. Negative controls included: (i) DacA-luciN (N-terminal luciferase domain alone fused to DacA), (ii) 723-luciC (C-terminal domain alone fused to SMU_723), and (iii) DacA-luciN + luciC (C-terminal domain expressed alone without a fusion partner). Luminescence signals were normalized to optical density at 600 nm (OD_600_). Data from three independent assays, each performed with three technical replicates, are presented as mean ± SD and were analyzed using an unpaired Student’s *t*-test (*P < 0.05; **P < 0.01; ***P < 0.001; and ****P < 0.0001).

Our mass spectrometry data suggest that DacA may participate in coordinated regulatory networks through interactions with proteins of diverse functional categories. To validate these interactions *in vivo*, we employed a split luciferase complementation assay (SLCA) and selected several candidates with differing levels of enrichment from the proteomic analysis, including SMU_723, FruI, Ftf, SecA, and GapC. The N-terminal domain of *Renilla* luciferase (amino acids 1–155) was fused to the C-terminus of DacA, while the C-terminal domain (amino acids 156–314) was fused to the C-terminus of each candidate interactor, generating strains such as DacA-luciN + 723-luciC, DacA-luciN + FruI-luciC, DacA-luciN + Ftf-luciC, DacA-luciN + SecA-luciC, and DacA-luciN + GapC-luciC. As a positive control, we constructed a DacA-luciN + GlmM-luciC fusion strain using GlmM (SMU_1426c), a known interactor of DacA. To account for non-specific interactions, we included several negative controls: (i) DacA-luciN, expressing only the N-terminal luciferase domain fused to DacA; (ii) 723-luciC, expressing only the C-terminal luciferase domain fused to SMU_723; and (iii) DacA-luciN + luciC, in which the luciferase C-terminal domain was expressed alone without a fusion partner. The SLCA results indicated that DacA specifically interacts with most of the tested candidates *in vivo*, including SMU_723, FruI, SecA, and GapC, as shown by their increased luminescence signals (Fig. 2). Of note, the DacA-luciN + Ftf-luciC strain did not exhibit a significantly increased luminescence signal compared to the DacA-luciN + luciC negative control, suggesting a possible indirect or weak interaction between DacA and Ftf.

### Assessment of cell morphology following deletion of DacA-interacting proteins

**Fig 3:**
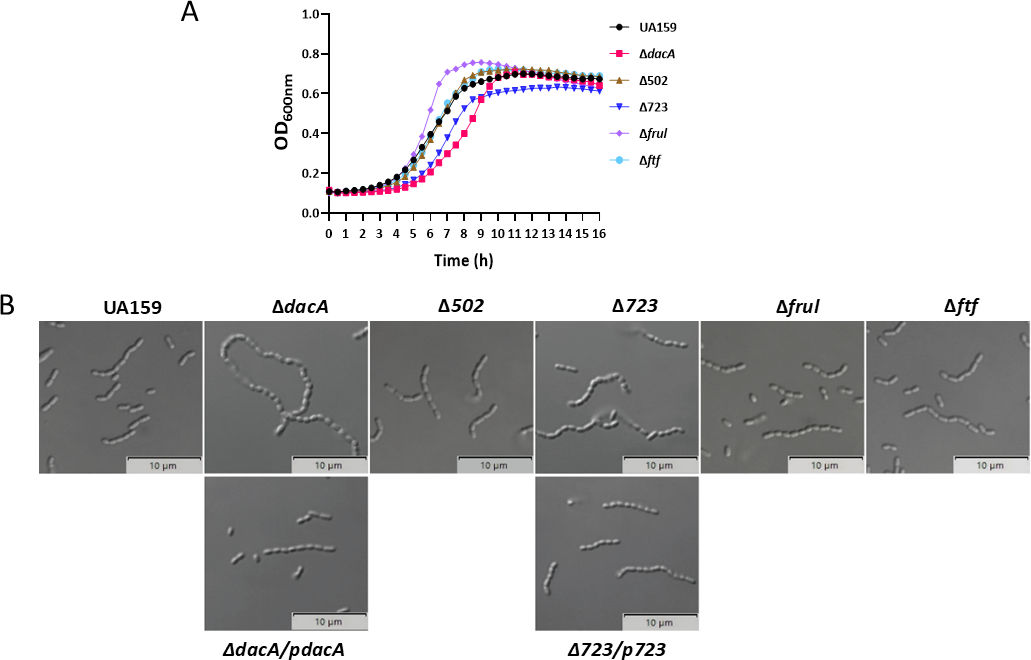
Growth and cell morphology changes in deletion strains of *S. mutans*. (**A**) Growth curves of wild-type strain UA159 and deletion mutants (Δ*dacA*, Δ502, Δ723, Δ*fruI,* and Δ*ftf*) were measured in THB medium using a 96-well plate format. The figure shows one representative result from three independent experiments; each performed with three technical replicates. (**B**) Cell morphology of overnight cultures observed under 100× oil immersion microscopy. Scale bar = 10 µm.

To explore the functional significance of DacA and its potential interacting partners, we selected several highly enriched candidates—SMU_502, SMU_723, FruI, and Ftf—for gene deletion analysis. We generated the corresponding mutants (Δ502, Δ723, Δ*fruI*, and Δ*ftf*) and included a previously characterized *dacA* deletion strain (Δ*dacA*) (13) for comparative phenotypic analysis. By examining these mutants, we aimed to identify shared phenotypic traits that might reflect functional connections between DacA and its interacting proteins. Growth curve analysis revealed that the Δ*dacA* strain exhibited a markedly prolonged lag phase, while the Δ723 mutant showed a moderately extended lag phase compared to the wild-type strain UA159 (Fig. 3A). In contrast, Δ502 and Δ*ftf* mutants displayed growth rates comparable to the wild-type strain, whereas Δ*fruI* exhibited an accelerated growth rate. These findings suggest that both DacA and SMU_723 positively contribute to growth, while FruI may be involved in a rate-limiting step in growth kinetics. Microscopic analysis further revealed distinct morphological abnormalities in the Δ*dacA* and Δ723 strains. When cultured in THB liquid medium, both mutants exhibited irregular cell shapes, in contrast to the uniform rod-like morphology observed in wild-type *S. mutans* UA159 and the other deletion strains (Fig. 3B). Notably, cell morphology was restored in the complemented strains, confirming that the observed defects were specifically due to the loss of the respective genes. These observations indicate that DacA and SMU_723 play important roles in maintaining proper cell morphology.

### Functional role of DacA interactors in biofilm formation

**Fig 4:**
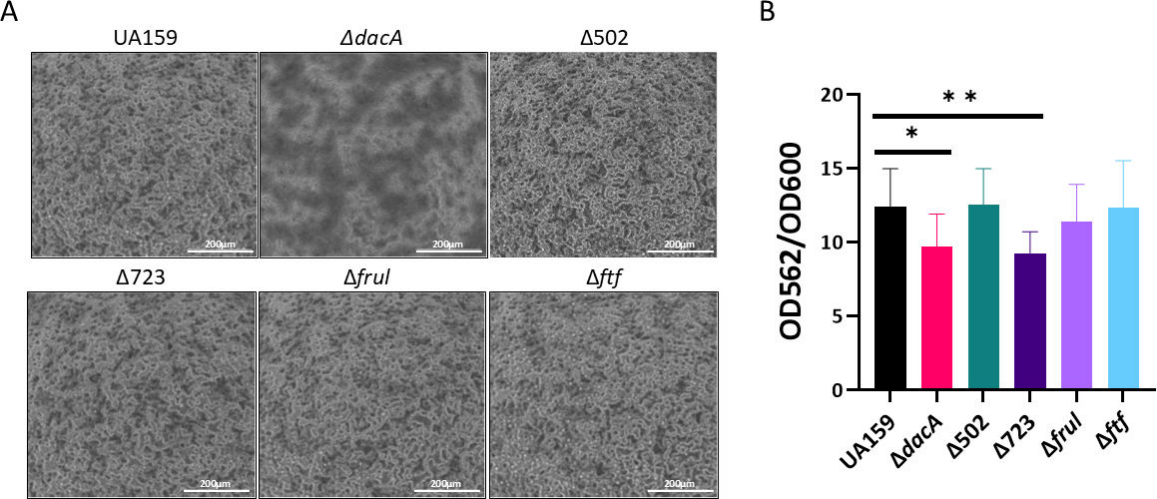
Biofilm formation and quantification. (**A**) Representative phase-contrast images of biofilms formed by wild-type strain UA159 and deletion mutants (Δ*dacA*, Δ502, Δ723, Δ*fruI*, and Δ*ftf*) grown in THB medium supplemented with 1% sucrose. Images were captured using a Cytation 5 Cell Imaging Multimode Reader at 40× magnification. Scale bar = 200 µm. (**B**) Biofilm biomass was quantified by crystal violet staining. Absorbance at 562 nm was normalized to OD_600_ values. The figure shows one representative result from three independent experiments, each performed with three technical replicates. Data are presented as mean ± SD and were analyzed using an unpaired Student’s *t*-test (*P < 0.05; **P < 0.01; ***P < 0.001; and ****P < 0.0001).

Given that biofilm formation is a key aspect of *S. mutans* pathogenicity and that the Δ*dacA* strain exhibits significant biofilm phenotype, we next investigated how deletion of DacA-interacting candidates affects this phenotype. Our results showed that only the Δ*dacA* strain formed biofilms with a markedly altered pattern compared to the wild-type and other mutant strains (Δ502, Δ723, Δ*fruI*, and Δ*ftf*) (Fig. 4A). Crystal violet staining further revealed that both Δ*dacA* and Δ723 mutants produced significantly less biomass relative to the wild-type strain, while no significant differences were observed in the Δ502, Δ*fruI*, and Δ*ftf* mutants (Fig. 4B). These findings suggest that DacA and SMU_723 play important roles in supporting robust biofilm formation in *S. mutans*.

### Functional impact of DacA interactors on acid resistance

**Fig 5:**
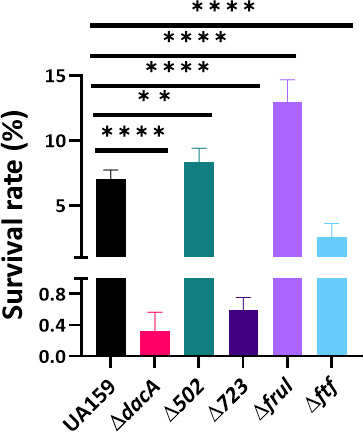
Acid shock response. Acid resistance of *S. mutans* wild-type strain UA159 and its deletion mutants (Δ*dacA*, Δ502, Δ723, Δ*fruI*, and Δ*ftf*) were assessed by exposing mid-log phase cultures to 0.1 M glycine-HCl buffer (pH 2.8) or control buffer (pH 7.0) for 5 min at 37°C, followed by dilution, plating on THB agar, and colony enumeration after 48 h of incubation. Survival rates were calculated as the ratio of colony-forming units (CFUs) from the acid-treated samples to the pH 7.0 control. The figure shows one representative result from three independent experiments, each performed with three technical replicates. Data are presented as mean ± SD and were analyzed using an unpaired Student’s *t*-test (*P < 0.05; **P < 0.01; ***P < 0.001; and ****P < 0.0001).

*S. mutans* is an aciduric bacterium capable of surviving and thriving in low-pH environments. To further investigate the role of DacA and its interacting partners in acid stress response, we examined acid tolerance in the deletion mutants. Log-phase cultures were exposed to an acid shock treatment using 0.1 M glycine-HCl buffer at pH 2.8. Following this exposure, the Δ*dacA*, Δ723, and Δ*ftf* strains exhibited significantly reduced viable cell counts compared to the wild-type strain UA159 (Fig. 5), indicating impaired survival under acidic conditions. Interestingly, the Δ502 and Δ*fruI* mutants showed significantly increased viable cell counts relative to UA159 (Fig. 5), suggesting a potential repressive role for these genes in acid stress adaptation. These findings suggest that DacA, SMU_723, and Ftf are involved in promoting *S. mutans* acid resistance. The enhanced survival of Δ502 and Δ*fruI* mutants under acid stress also raises the possibility that deletion of these genes may relieve negative regulation of acid tolerance mechanisms.

### Functional impact of DacA interactors on sugar utilization

**Fig 6:**
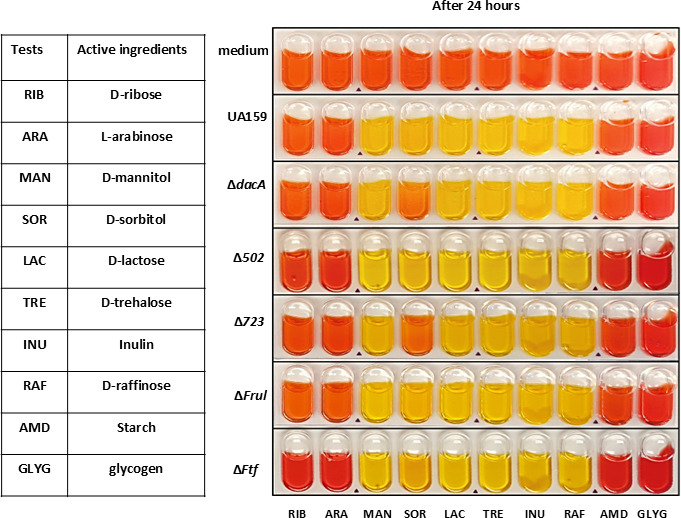
Sugar utilization profiling. Metabolic activity of *S. mutans* wild-type strain UA159 and deletion mutants (Δ*dacA*, Δ502, Δ723, Δ*fruI*, and Δ*ftf*) was assessed at 24-h time point using the API 20 Strep system. Medium alone served as a negative control to determine which sugars were metabolized by bacterial strains. The figure shows one representative result from three independent experiments.

Given that *S. mutans* utilizes a wide range of carbohydrates to produce acid and form biofilms, we investigated whether deletion of *dacA* and SMU_723 impacts sugar metabolism. To assess this, we performed sugar utilization profiling using the API 20 Strep system. Both the Δ*dacA* and Δ723 mutants showed significantly impaired sorbitol metabolism at the 24-h time point compared to the wild-type strain, suggesting a delayed metabolic response (Fig. 6). By contrast, the Δ502, Δ*fruI*, and Δ*ftf* mutants did not exhibit noticeable differences in sugar utilization. These findings indicate that DacA and SMU_723 are important for efficient sorbitol metabolism, which may be essential for rapid environmental adaptation and the pathogenic potential of *S. mutans* in the oral cavity.

### Deletion of *dacA* or SMU_723 reduces acid production

**Fig 7:**
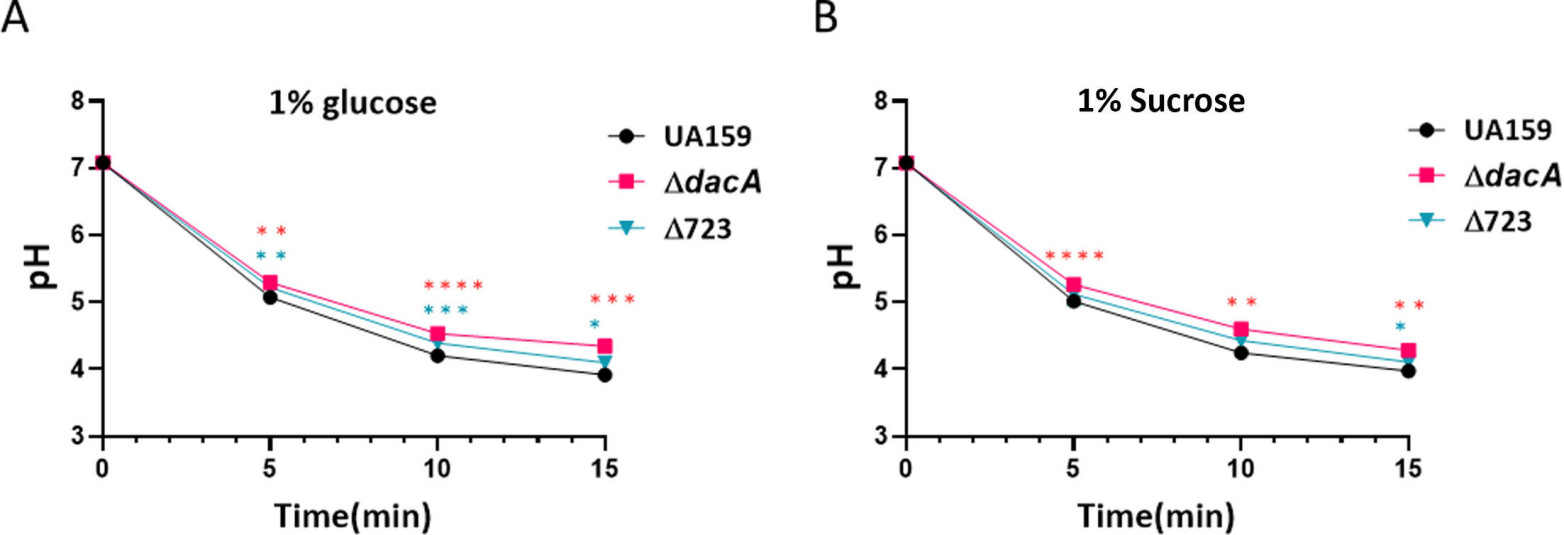
Glycolytic activity. Glycolytic pH drop assays were performed using 1% glucose (**A**) and 1% sucrose (**B**) as substrates. *S. mutans* wild-type strain UA159 and deletion mutants (Δ*dacA* and Δ723) were assessed. pH measurements over time were analyzed using two-way ANOVA followed by Tukey’s post hoc test. The figure shows one representative result from three independent experiments, each with three technical replicates. Data are presented as mean ± SD (*P < 0.05; **P < 0.01; ***P < 0.001; and ****P < 0.0001).

Given the substantial overlap in phenotypic and functional changes observed in the ΔdacA and Δ723 mutants, we sought to further investigate the biological role of SMU_723 in relation to DacA in acid production. We performed a glycolytic pH drop assay using 1% glucose and 1% sucrose as substrates. The wild-type strain UA159 exhibited the most rapid and pronounced pH decrease within the first 15 min. In contrast, the Δ723 mutant showed a moderate reduction in pH, while the Δ*dacA* strain displayed the least pH drop (Fig. 7A and B). These results suggest that both DacA and SMU_723 contribute to acid production to varying extents.

### Deletion of *dacA* or SMU_723 reduces *S. mutans* colonization in *Drosophila melanogaster*

**Fig 8:**
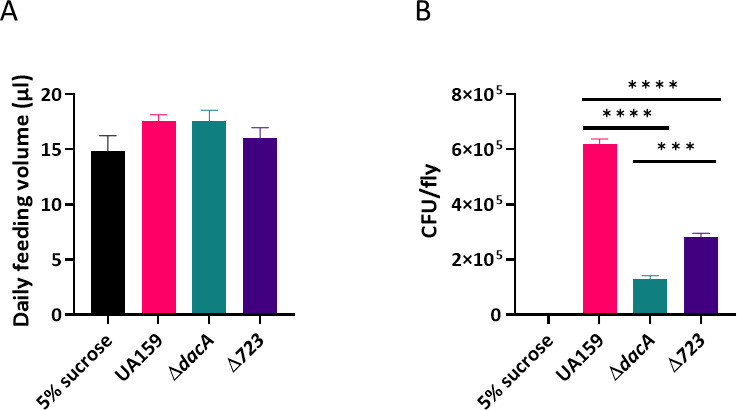
Bacterial colonization in *Drosophila in vivo. S. mutans* wild-type strain UA159 and its deletion mutants (Δ*dacA* and Δ723) were assessed. (**A**) Daily feeding volume per group was measured over 3 days (nine flies per group). (**B**) Bacterial colonization levels were determined by colony-forming unit (CFU) counts. For each group, samples were plated in triplicate to assess variation. Data are presented as mean ± SD and were analyzed using an unpaired Student’s *t*-test (*P < 0.05; **P < 0.01; ***P < 0.001; and ****P < 0.0001). The figure shows one representative result from three independent experiments.

To evaluate the role of DacA and SMU_723 in bacterial colonization *in vivo*, we used *Drosophila melanogaster* as a model host for colonization. Flies from all experimental groups consumed comparable amounts of 5% sucrose, with or without bacterial supplementation, over a 3-day feeding period. Despite similar intake, colonization levels were significantly lower in flies fed with either the Δ*dacA* or Δ723 mutant strains compared to those fed with the wild-type strain (Fig. 8A and B). These results indicate that both DacA and SMU_723 are important for effective colonization of *S. mutans in vivo*.

### Threonine 147, glutamine 148, and threonine 149 in *S. mutans* DacA are critical for interaction with SMU_723, and disruption of this interaction alters intracellular c-di-AMP production

**Fig 9:**
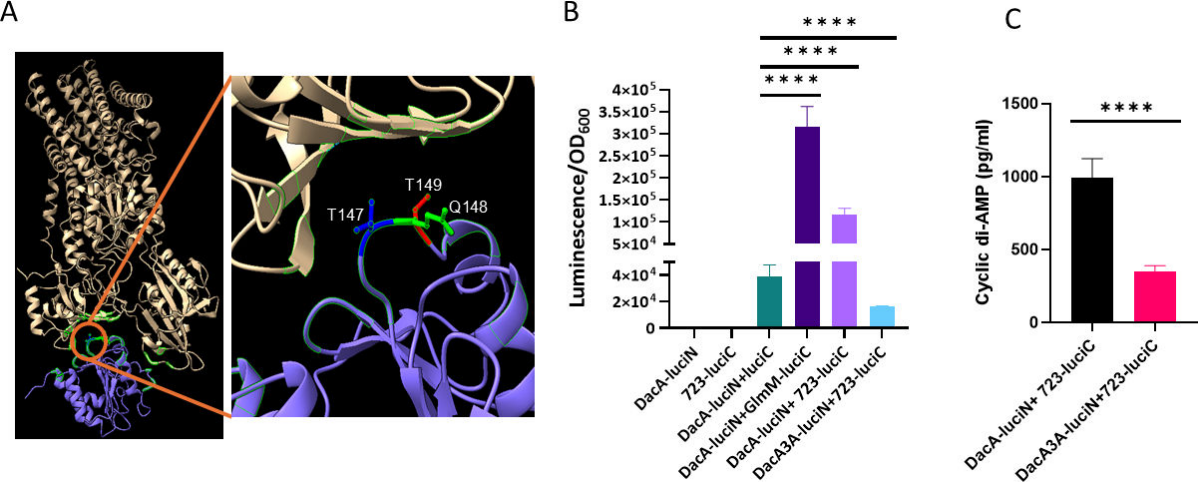
Prediction and validation of critical residues mediating the DacA–SMU_723 protein interaction. (**A**) The interaction between DacA (excluding its transmembrane domain) and full-length SMU_723 was predicted using AlphaFold3 (23). The resulting structural model was visualized with UCSF ChimeraX version 1.9 (24). Three residues in DacA—threonine 147 (T147), glutamine 148 (Q148), and threonine 149 (T149)—were identified at the predicted interface and are highlighted. (**B**) To assess the functional importance of these residues, a site-directed mutant DacA3A-luciN + 723-luciC was generated by substituting T147, Q148, and T149 with alanine. This mutant was tested alongside the wild-type interaction pair DacA-luciN + 723-luciC, a positive control DacA-luciN + GlmM-luciC, and three negative controls: (i) DacA-luciN, (ii) 723-luciC, and (iii) DacA-luciN co-expressed with unfused luciC. Luminescence signals were normalized to OD_600_. The figure shows one representative result from three independent experiments, each performed with three technical replicates. Data are presented as mean ± SD and were analyzed using an unpaired Student’s *t*-test (*P < 0.05; **P < 0.01; ***P < 0.001; and ****P < 0.0001). (**C**) c-di-AMP production was measured in the site-directed mutant strain DacA3A-luciN + 723-luciC and the parental strain DacA-luciN + 723-luciC using the Cayman c-di-AMP detection kit. The figure presents combined data from three independent biological replicates; each performed with three technical replicates. Data are shown as mean ± SD and were analyzed using an unpaired Student’s *t*-test (*P < 0.05; **P < 0.01; ***P < 0.001; and ****P < 0.0001).

To further validate the in *vivo* interaction between DacA and SMU_723, we used AlphaFold3 to predict the structure of their protein complex (Fig. 9A). The model suggested a potential interaction interface involving residues threonine 147, glutamine 148, and threonine 149 in DacA. To assess the functional importance of these residues, we generated a DacA alanine substitution variant (DacA3A), in which T147, Q148, and T149 were replaced with alanine. This mutant was fused to the luciferase N-terminal domain (amino acids 1–155) and paired with the luciferase C-terminal domain (amino acids 156–314) fused to the C-terminus of SMU_723, generating the DacA3A-luciN + 723-luciC strain. Luminescence from the DacA3A-luciN + 723-luciC strain was significantly reduced compared to the DacA-luciN + 723-luciC strain (Fig. 9B), indicating that substitution of these residues disrupts the interaction. Consistent with this disruption, the DacA3A mutant also exhibited a significant decrease in intracellular c-di-AMP levels relative to the parental strain (Fig. 9C). Together, these results demonstrate that threonine 147, glutamine 148, and threonine 149 are critical for DacA–SMU_723 binding and that this interaction contributes to the regulation of c-di-AMP production.

### DacA–SMU_723 interaction modulates calcium-dependent growth in *S. mutans*

**Fig 10:**
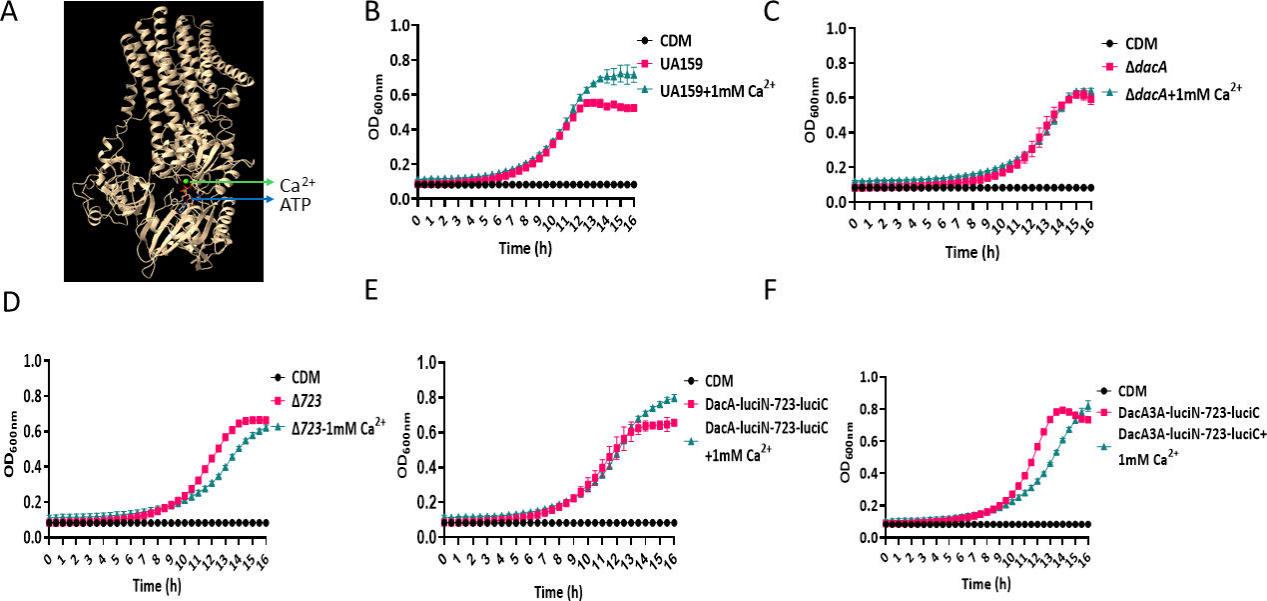
Structural prediction of SMU_723 as a calcium transporter and assessment of calcium involvement in bacterial growth regulation. (**A**) The full-length SMU_723 protein structure in complex with Ca^2+^ and ATP was predicted using AlphaFold3 (23). Growth curves of wild-type strain UA159 (**B**), deletion strain ΔdacA (**C**), Δ723 strain (**D**), DacA-luciN + 723-luciC strain (**E**), and site-mutated strain DacA3A-luciN + 723-luciC (**F**) were measured in chemically defined media with or without 1 mM calcium. Bacteria were cultured in a 96-well plate, and the plate was incubated in the Cytation 5 Cell Imaging Multimode Reader at 37°C with 5% CO_2_. Optical density at 600 nm (OD_600_) was recorded over time. The figure presents a representative result from three independent assays; each performed with three technical replicates. Data are presented as mean ± SD.

SMU_723 encodes a predicted transmembrane protein homologous to the calcium ATPase LMCA1 from *Listeria monocytogenes*, sharing 65% amino acid identity. Recent structural studies have demonstrated that LMCA1 plays an essential role in calcium transport in *L. monocytogenes* (25). Using AlphaFold3, we predicted the structure of SMU_723 in complex with Ca^2+^ and ATP, revealing a conformation similar to that of LMCA1 (Fig. 10A). Given the direct interaction between DacA and SMU_723 identified in our assays, we hypothesized that calcium signaling may be an important component of the DacA regulatory network. To investigate this, we assessed bacterial growth in response to CaCl_2_ supplementation. The wild-type *S. mutans* UA159 strain exhibited enhanced growth in the presence of 1 mM calcium (Fig. 10B). In contrast, the Δ*dacA* mutant showed no enhanced growth upon calcium supplementation (Fig. 10C), suggesting a loss of calcium responsiveness. The Δ723 mutant displayed a prolonged lag phase and a slightly reduced growth rate in the presence of calcium (Fig. 10D), indicating that SMU_723 is also required for calcium-dependent growth enhancement. To further explore the functional significance of the DacA–SMU_723 interaction, we tested calcium effects on strains expressing fusion proteins used in our SLCA assay: one strain (DacA-luciN + 723-luciC) contains intact DacA and SMU_723 fusion proteins, while the other (DacA3A-luciN + 723-luciC) harbors a site mutation in DacA that disrupts its interaction interface with SMU_723. The DacA-luciN + 723-luciC strain exhibited enhanced growth in the presence of calcium, similar to the wild type (Fig. 10E). In contrast, the DacA3A-luciN + 723-luciC strain showed a prolonged lag phase and a slightly reduced growth rate upon calcium supplementation, closely resembling the Δ723 phenotype (Fig. 10F). These findings suggest that disruption of the DacA–SMU_723 interaction weakens calcium responsiveness.

## DISCUSSION

By combining affinity purification under both non-crosslinked and crosslinked conditions with mass spectrometry-based proteomics, we captured a broad spectrum of stable and transient DacA interactions. Robust enrichment of the known partners CdaR and GlmM in our data set internally validates our experimental approach and reaffirms conserved, operon-linked regulation of DacA associated activity. Building on this foundation, we delineated a more complex DacA protein-protein interaction network. We identified constitutive partners present in both conditions—SMU_723, FruI, FtsH, SecA, PttB, and Pbp1a. Additionally, we also identified interactions preferentially detected after crosslinking-SMU_502, FtsK, SMU_1412c, OpuCa, PknB, EzrA, MltG, ComA, DivIVA, KhpB, and FtsA. The differential recovery across conditions underscores the value of orthogonal capture strategies to reveal the full spectrum of protein interactions.

Functionally, the network is strikingly diverse. Identification of enzymes of central metabolism, including GlmS, GuaB, GapC, GlmM, Ftf, and GtfBin the network, suggests that DacA sits at the intersection of second messenger signaling and metabolic flux. The prominence of transport-related proteins, multiple ABC transporter components (e.g., SMU_723, SloA, LivF, LivG, SMU_1412c, OpuCa, and ComA) and PTS elements (e.g., FruI and PttB) is consistent with c-di-AMP’s established role in controlling ion/osmolyte homeostasis and turgor (11, 26). Identification of cell division and morphogenesis factors like FtsZ, FtsA, EzrA, DivIVA, PknB, and Pbp1a implicates DacA in coordinating envelope growth with cell-cycle progression. Consistent with this, c-di-AMP functions as a master regulator of cell volume and turgor, coupling osmotic homeostasis with cell wall integrity (11, 27). Interactions with protein quality control/trafficking machinery (GroEL, FtsH, and SecA) raise the possibility that DacA activity or turnover is tuned by proteostasis. Finally, interactions with nucleic acid-associated proteins (e.g., RecA, FtsK, and KhpB) hint at c-di-AMP-linked responses to DNA damage, repair, and RNA metabolism. Together, these observations extend DacA’s influence well beyond c-di-AMP synthesis *per se*, positioning it as a signaling hub that integrates transport, envelope homeostasis, and growth. A particularly notable finding is the DacA interaction with OpuCa, an osmolyte transporter component whose orthologs are established c-di-AMP receptors in multiple bacterial species, including *S. agalactiae* (11), *Listeria monocytogenes*, *Enterococcus faecalis* (28), *Staphylococcus aureus* (29), and *Bacillus subtilis* (30). This dual connectivity-protein-protein association with DacA and ligand recognition of c-di-AMP supports a feedback loop or co-regulation mechanism in which c-di-AMP both (i) modulates transporter activity and (ii) is, in turn, regulated by physical proximity or scaffolding with transport machinery via tuned DacA activity. Such reciprocal coupling could stabilize intracellular osmotic balance by matching second messenger production to the status of osmolyte flux, a regulatory motif that has been proposed in other gram-positive systems (4, 30).

Because of its strong, reproducible enrichment and conservation, we focused on SMU_723, a predicted P-type ATPase. It shows remarkable conservation across *Streptococci*, maintaining 72–81% amino acid sequence identity. Notably, it also shares significant homology to LMCA1 in *L. monocytogenes* (65% identity), a known calcium exporter critical for bacterial survival under calcium stress, supporting its role in calcium efflux. Multiple lines of evidence link SMU_723 functionally to DacA: (i) Δ723 exhibits several defects also observed in the Δ*dacA* strain, including a prolonged lag phase, altered cell morphology, reduced acid production and acid tolerance, impaired sorbitol metabolism, and diminished colonization in the *Drosophila* model. Notably, these phenotypes are consistently less severe than those of the Δ*dacA*, indicating partial functional overlap rather than complete equivalence. In addition, both the Δ*dacA* and Δ723 mutants displayed defects in sorbitol metabolism, further supporting their involvement in the same regulatory or metabolic pathway. The mechanism underlying this phenotype remains unclear. It is not yet known whether the sorbitol PTS system is directly regulated by c-di-AMP in *S. mutans*, or whether the defect reflects a broader fitness cost or osmotic imbalance resulting from disrupted c-di-AMP homeostasis. Additional biochemical studies will be required to determine whether this represents a specific regulatory link or a more general physiological consequence. While the Drosophila model provides a convenient *in vivo* system to assess bacterial colonization and viability, it differs substantially from the human oral environment in oral anatomy, salivary flow, and microbiota composition. Thus, these findings may not fully represent bacterial behavior in the human oral cavity. (ii) Both mutants failed to mount calcium-stimulated growth responses. (iii) Alanine substitutions at Thr147, Gln148, Thr149 within DacA’s catalytic domain—residues predicted by AlphaFold to contact SMU_723—attenuate binding and recapitulate delayed growth onset upon Ca^2+^ supplementation. These convergent phenotypes argue that the DacA–SMU_723 interaction promotes timely adaptation to environmental cues, with calcium emerging as a key input. In contrast to calcium supplementation, neither Mg^2+^ (Fig. S1A through E) nor Mn^2+^ (Fig. S2A through E) produced a comparable effect. These findings indicate that the observed phenotype is not attributable to a general divalent cation or ionic strength effect but is instead specific to calcium. This further supports the proposed role of SMU_723 as a calcium-specific ATPase involved in calcium homeostasis. From these results, we propose three non-exclusive regulatory scenarios: (i) DacA to Smu_723 control: DacA regulates SMU_723 activity or localization, coupling c-di-AMP production to calcium export activity and envelope physiology; (ii) SMU_723-dependent control of DacA: Smu_723 modulates DacA (e.g., by scaffolding, lipid-microdomain positioning, or conformational control) to adjust c-di-AMP output in response to calcium activities; or (iii) Integrated hub: DacA, Smu_723, and OpuC/other transporters assemble into a novel signaling complex that coordinates c-di-AMP and calcium signaling pathways to balance turgor, cell-wall biogenesis, and growth. Future studies in mechanistic dissection of the DacA-Smu_723 interface should clarify the working model. In conclusion, our findings position DacA as a central node in *S. mutans* that coordinates second messenger signaling with ion transport, metabolism, and cell cycle processes. The DacA–SMU_723 axis, together with OpuCa and additional transport/division partners, suggests a membrane-embedded regulatory network that couples c-di-AMP dynamics to calcium and osmotic homeostasis. The fact that both a dacA and SMU_723 deletion led to a delayed sorbitol metabolism is a strong indication that they are part of the same pathway. The observed delay suggests that without one or both components, the bacterium loses its ability to rapidly “turn on” or fully activate its sorbitol metabolic machinery. Investigating how these interactions shape virulence traits (biofilm formation, acid tolerance, and bacterial colonization) may uncover new targets for selectively disrupting cariogenic physiology while minimizing impacts on commensals.

## MATERIALS AND METHODS

### Bacterial strains and culture conditions

The bacterial strains and plasmids used in this study are listed in Table S1, and S. *mutans* UA159 served as the wild-type parental strain. All *S. mutans* strains were cultured in Todd-Hewitt medium (Difco) or chemically defined medium (31) and incubated at 37°C in a 5% CO_2_ environment. For the selection of antibiotic-resistant colonies, THB plates were supplemented with 1 mg/mL spectinomycin, 800 µg/mL kanamycin, and 12.5 µg/mL erythromycin.

### DNA manipulation and strain construction

Primers used in this study are listed in Table S2. Specific details of strain construction are described in the supplemental material. Individual polymerase chain reactions (PCRs) were performed using KOD Hot Start DNA Polymerase (Millipore Sigma), while overlap extension PCR (OE-PCR) was performed using KOD Xtreme Hot Start DNA Polymerase (Millipore Sigma).

### Co-immunoprecipitation

Co-immunoprecipitation assay was performed as previously described (32). Briefly, *S. mutans* strains UA159 and DAC-FLAG were cultured at 37°C in a CO_2_ incubator to mid-log phase in THB medium, with 12.5 µg/mL erythromycin added for DacA-FLAG. Cells were pelleted, washed with ice-cold phosphate-buffered saline (PBS), and divided into two groups: non-crosslinked and crosslinked samples. For crosslinked samples, cells were incubated in PBS containing 1% (vol/vol) formaldehyde for 20 min at room temperature, followed by quenching with 0.125 M glycine for 5 min. After washing and pelleting, cells were sonicated in lysis buffer (150 mM NaCl, 10% [vol/vol] glycerol, 100 mM Na-phosphate buffer [pH 7.0] and 1% Triton X-100, protease inhibitor cocktail, pH 7.0), and the supernatant obtained after cell lysis was incubated with anti-FLAG M2 affinity gel (Sigma-Aldrich) for 3 h at 4°C with end-over-end rotation. FLAG Peptide (Sigma) with a working concentration of 150 ng/µL was used for the competitive elution of FLAG tagged DacA. The wild-type strain UA159 served as a no-FLAG epitope control sample.

### In-gel digestion and LC-MS/MS analysis

Proteins were separated on NuPAGE 10% bis-tris gels (Invitrogen) at 150 V for 5 min and stained with Coomassie Blue. Visible bands were excised, cut into ~1 mm^2^ pieces, and subjected to in-gel digestion using Trypsin Gold (Promega) and ProteaseMAX surfactant (Promega) following the manufacturer’s protocol. Mass spectrometry analysis was conducted at the Oregon Health & Science University (OHSU) Proteomics Shared Resource.

### Split luciferase complementation assays

Split luciferase complementation assays were performed as previously described (33). Overnight *S. mutans* cultures were diluted 1:20 in THB and grown to mid-log phase. Luciferase activity was measured after adding 1 µL of 0.75 mg/mL coelenterazine-h solution (Fisher) to 100 µL of cell culture using a GloMAX Discover 96-well luminometer (Promega). To normalize the data, luciferase values were divided by their corresponding optical density (OD_600_) values. The experiment was performed in triplicate and repeated three times.

### Growth curve measurement

Overnight cultures of *S. mutans* strains were diluted 1:20 in fresh THB and grown to an OD_600_ of 0.4. The cultures were then further diluted 1:100 in fresh THB and incubated in a 96-well plate at 37°C with CO_2_ using a Cytation 5 Cell Imaging Multimode Reader. Cell growth was monitored at OD_600_ every 30 min, with a 10-s shaking step at 205 cpm before each reading to ensure proper mixing. To investigate the effects of calcium on cell growth, chemically defined medium was used in place of THB, and CaCl_2_ was added to a final concentration of 1 mM. The experiment was performed in triplicate and repeated three times.

### Cell morphology examination

Cell morphologies of *S. mutans* strains were examined and imaged using an Olympus IX73 inverted microscope equipped with a 100× oil immersion objective.

### Biofilm imaging and quantification

*S. mutans* strains were cultured overnight in THB medium, subcultured into fresh THB to mid-log phase. Cells were washed with 1× PBS and adjusted to an OD_600_ of 0.4 in 1× PBS. Cultures were then diluted 1:1,000 into THB supplemented with 1% sucrose and inoculated into a 96-well plate. The plate was incubated for 16 h at 37°C in a 5% CO_2_ incubator. Following incubation, OD_600_ was measured. Then, the plate was gently washed three times with 1× PBS. Biofilm morphology was then imaged using the Cytation 5 Cell Imaging Multimode Reader at 40× phase contrast. After that, biofilms were stained with 0.1% crystal violet. After washing with 1× PBS, the bound dye was solubilized with 30% acetic acid. Absorbance was measured at 562 nm and normalized to OD_600_ values. The experiment was performed in triplicate and independently repeated three times.

### Glycolytic pH drop assay

The glycolytic pH drop assay was performed as previously described (34). *S. mutans* strains were grown overnight in THB medium and subcultured until reaching an OD_600_ of 0.6. Cells were harvested, washed, and resuspended in a solution containing 50 mM KCl and 1 mM MgCl_2_, adjusted to pH 7.0. Glucose and sucrose were added to the cell suspension at a final concentration of 1%, and the pH was measured every 5 min for 15 min at room temperature using a Fisherbrand accumet AB15 Basic pH meter. The experiment was performed in triplicate and repeated three times.

### Acid shock assay

*S. mutans* acid tolerance was evaluated as previously described (34). *S. mutans* strains were cultured overnight in THB medium and subsequently subcultured until reaching an OD_600_ of 0.6. Cells were then pelleted, washed with 1× PBS, and resuspended in 0.1 M glycine-HCl buffer at either pH 2.8 (acid treatment) or pH 7.0 (control). The suspensions were incubated at 37°C for 5 min, followed by dilution in 1× PBS and plating on THB agar. After incubation at 37°C for 48 h, colonies were counted, and the survival rate was calculated by dividing the colony-forming units (CFUs) from the acid-treated group by those from the pH 7.0 control. The experiment was performed in triplicate and repeated three times.

### Sugar metabolism test

The API 20 Strep system was used to assess the metabolic profiling of sugar utilization in *S. mutans* strains. Briefly, overnight cultures of *S. mutans* strains were subcultured in THB medium until reaching mid-log phase. Cells were then pelleted, washed with 1× PBS, and resuspended in the API 20 Strep medium to an OD_600_ of 0.4. The medium alone served as a negative control. The strips were incubated at 37°C in a CO_2_ incubator, and metabolic activity was recorded after 24 h. The experiment was repeated three times.

### *In vivo* colonization assay

*Drosophila* Canton-S was used as an *in vivo* model to assess bacterial colonization, following a modified version of previously described methods (35–37). Briefly, healthy 3-day-old male flies were pretreated with antibiotics for 2 days, then grouped into vials (nine flies per vial). Flies were fed twice daily for 3 days with either 5% sucrose (control) or 5% sucrose containing *S. mutans* strains, delivered via capillaries (Fisher #NC127687). At the end of the feeding period, flies were homogenized in 200 µL of 1× PBS using a plastic pestle. Serial dilutions were plated on THB agar and incubated at 37°C for 48 h for colony enumeration. The assay was conducted under a 12-h light/dark cycle at 25°C in a *Drosophila* incubator and repeated three times.

### Data analysis

Statistical analyses were performed using Prism 10 (GraphPad Software Inc.). An unpaired two-tailed Student’s *t*-test was used for comparisons between two groups. For comparisons involving multiple time points, two-way ANOVA followed by Tukey’s post hoc test was applied. A *P* value < 0.05 was considered statistically significant. Data are presented as mean ± SD.

## Data Availability

The UA159 *Streptococcus mutans* reference genome is publicly available in NCBI (RefSeq accession number: NC_004350.2). Summarized mass spectrometry data are presented in Fig. 1B and C. All other data supporting the findings of this study are available from the corresponding author upon request.

## SUPPLEMENTAL MATERIAL

Supplemental material msystems.01727-25-s0001.pdf Supplemental text, tables, and figures.

ASM does not own the copyrights to Supplemental Material that may be linked to, or accessed through, an article. The authors have granted ASM a non-exclusive, world-wide license to publish the Supplemental Material files. Please contact the corresponding author directly for reuse.
