# Supplementary material for: Decoding the interactome for cyclic-di-AMP-producing enzyme diadenylate cyclase
Source: mSystems. 2026 Jun 3;11(6):e01727-25. doi: 10.1128/msystems.01727-25 (PMC13289029; doi:10.1128/msystems.01727-25)
Supplement: Supplemental material — Supplemental text, tables, and figures. [file msystems.01727-25-s0001.pdf]

Supplemental Experimental Procedures

Construction of a C-terminal FLAG-Tagged DacA strain for Co-Immunoprecipitation analysis

A FLAG tag (GATTACAAGGACGATGACGATAAG) was fused to the C-terminus of DacA, with a flexible GGGGS linker inserted between DacA and the FLAG tag. To construct this fusion, the 5′ flanking region was first amplified using primers 14285fF/R to append the GGGGS linker to the end of the *dacA* gene. This product was then used as a template for a second PCR with primers 14285fF/R2 to add the FLAG tag sequence. In parallel, the 3′ flanking region was amplified using primers 14283fF/R to insert a ribosomal binding site upstream of the *SMU_1427c* open reading frame. This fragment was subsequently used as a template in a second PCR with primers 14283fF2/R to introduce an overlapping sequence with the 5′ flanking region. The resulting 5′ and 3′ flanking fragments, containing complementary overlapping regions, were assembled via overlap extension PCR (OE-PCR) using primer pair 14285fe3F/R. The final fusion construct was then transformed into the wild-type *Streptococcus mutans* strain UA159, and colony PCR screening was performed to identify DacA-FLAG strains containing the integrated FLAG tag.

Construction of deletion strains

Construction of the ΔSMU_502 deletion strain

An allelic exchange strategy was used to construct the ΔSMU_502 deletion strain. Briefly, the upstream and downstream flanking regions of SMU_502 were PCR-amplified using primer pairs 5025fF/R and 5023fF/R, respectively. An erythromycin resistance cassette (ermB) was also amplified using primers ErmF/R. Each of the three fragments contained overlapping sequences to facilitate assembly via overlap extension PCR (OE-PCR) using the primer pair 5025e3F/R. The resulting fusion product was then transformed into the wild-type *S. mutans* UA159 strain, and erythromycin-resistant colonies were selected to isolate the final Δ502 strain.

Construction of the ΔSMU_723 deletion strain

An allelic exchange strategy was used to construct the ΔSMU_723 deletion strain. Briefly, the upstream and downstream flanking regions of SMU_723 were PCR-amplified using primer pairs 7235fF/R and 7233fF/R, respectively. An erythromycin resistance cassette (ermB) was also amplified using primers ErmF/R. Each of the three fragments contained overlapping sequences to facilitate assembly via overlap extension PCR (OE-PCR) using the primer pair 7235e3F/R. The resulting fusion product was then transformed into the wild-type *S. mutans* UA159 strain, and erythromycin-resistant colonies were selected to isolate the final Δ723 strain.

Construction of the Δ*fruI* deletion strain

An allelic exchange strategy was used to construct the Δ*fruI* deletion strain. Briefly, the upstream and downstream flanking regions of *fruI* were PCR-amplified using primer pairs 8725fF/R and 8723fF/R, respectively. An erythromycin resistance cassette (ermB) was also amplified using primers ErmF/R. Each of the three fragments contained overlapping sequences to facilitate assembly via overlap extension PCR (OE-PCR) using the primer pair 8725e3F/R. The resulting fusion product was then transformed into the wild-type *S. mutans* UA159 strain, and erythromycin-resistant colonies were selected to isolate the final Δ*fruI* strain.

Construction of the Δ*ftf* deletion strain

An allelic exchange strategy was used to construct the Δ*ftf* deletion strain. Briefly, the upstream and downstream flanking regions of *fruI* were PCR-amplified using primer pairs 20285fF/R and 20283fF/R, respectively. An erythromycin resistance cassette (ermB) was also amplified using primers ErmF/R. Each of the three fragments contained overlapping sequences to facilitate assembly via overlap extension PCR (OE-PCR) using the primer pair 20285e3F/R. The resulting fusion product was then transformed into the wild-type *S. mutans* UA159 strain, and erythromycin-resistant colonies were selected to isolate the final Δ*ftf* strain.

Construction of complementary strains.

Construction of the Δ*dacA*/p*dacA* complementary strain

To generate the complemented strain for Δ*dacA*, the open reading frame (*dacA*) along with its native promoter was amplified from Streptococcus mutans UA159 genomic DNA using the primer pair 1428pF/R, which included terminal sequences complementary to the pVA380 vector. The pVA380 vector backbone was PCR-amplified from the pDL278 plasmid (Chen & LeBlanc, 1992) using the primers pVA380F/R. Both amplicons were assembled via the Gibson assembly method (Gibson, 2011). The assembled plasmid construct was transformed into the Δ*dacA* mutant strain, and transformants were selected on agar plates containing spectinomycin. The resulting complemented strain, ΔdacA/pdacA, carried the pdacA plasmid. The plasmid was extracted and verified by sequencing.

Construction of the Δ*723*/p*723* complementary strain

To construct the complemented strain for Δ723, the open reading frame (SMU_723) along with its native promoter region was amplified from *S. mutans* UA159 genomic DNA using the primer pair 723pF/R. The pVA380 vector backbone was amplified using the primer pair pVA380F/R. The resulting fragments were assembled using the Gibson assembly method, and the assembled product was transformed into the Δ723 mutant strain. Transformants were selected on spectinomycin-supplemented agar plates to obtain the complemented strain Δ723/p723, which harbors the p723 plasmid. The plasmid was subsequently extracted and sequenced for verification.

Construction of strains for split luciferase complementation assays

Construction of the DacA-luciN fusion strain

A partial open reading frame (ORF) of SMU_1428c was amplified as the 5′ flanking region using the primer pair 1428luc5fF/R. A flexible 3× GGGGS linker followed by the N-terminal domain (amino acids 1–155) of luciferase was amplified from the genomic DNA of strain RMRenG (Qin et al., 2021) using primers 1428lucF/R. An erythromycin resistance cassette (ermB) was amplified with the primer pair ErmF2/R2. The downstream region of SMU_1428c was amplified as the 3′ flanking region using primer pair 1428luc3fF/R, incorporating a ribosomal binding site upstream of the SMU_1427c open reading frame. These four fragments were assembled via overlap extension PCR (OE-PCR) using the primer pair 1428luc5e3F/R. The resulting fusion construct was transformed into the wild-type *S. mutans* UA159 strain, and erythromycin-resistant colonies were selected to generate the DacA-luciN strain.

Construction of the 723-luciC and DacA-luciN+723-luciC Fusion Strains

A partial open reading frame (ORF) of *SMU_723* was amplified as the 5′ flanking region using the primer pair 723luc5fF/R, while the downstream sequence of *SMU_723* was amplified as the 3′ flanking region using primer pair 723luc3fF/R. A separate PCR amplicon containing a flexible 3× GGGGS linker followed by the C-terminal domain of luciferase (amino acids 156–314) and a kanamycin resistance cassette (**aphAIII**) was amplified from the genomic DNA of strain MecA-NTD + GyrA-CTD (Qin et al., 2024) (Qin et al., 2024) using primer pair 723lucF/R. These three fragments were assembled via overlap extension PCR (OE-PCR) using the primer pair 723luc5k3F/R. The resulting

fusion product was transformed into both the *Streptococcus mutans* UA159 strain and the previously constructed DacA-luciN strain. Kanamycin-resistant colonies were selected to generate the 723-luciC strain and the double fusion strain DacA-luciN+723-luciC.

Construction of DacA3A-luciN+723-luciC strain

A partial open reading frame (ORF) of SMU_1428c, along with its upstream sequence, was PCR-amplified using primer pair 14283Aluc5fF/R. This initial fragment served as a template for a second PCR to introduce overlapping sequences using primer pair 14283Aluc5fF2/R2. Separately, a PCR amplicon containing the site-mutated ORF of SMU_1428c, followed by a flexible 3× GGGGS linker, the luciferase N-terminal domain (amino acids 1–155), and an erythromycin resistance cassette (ermB), was amplified from the genomic DNA of the DacA-luciN strain using primer pair 14283Aluc3fF/R. The two PCR products were assembled via overlap extension PCR (OE-PCR) using primer pair 14283Aluc5e3F/R. The final fusion construct was transformed into the *S. mutans* 723-luciC strain, and erythromycin-resistant colonies were selected to generate the DacA3A-luciN+723-luciC strain.

Construction of DacA-luciN+luciC strain

A partial open reading frame (ORF) of SMU_1200 was amplified as 5' flanking region using primer pair 12005fF/R, while its downstream sequence was amplified as 3' flanking region using primer pair 1200luc3fF/R. A third PCR fragment, containing a ribosomal binding site and start codon followed by a flexible 3× GGGGS linker, the luciferase C-terminal domain (amino acids 156–314), and a kanamycin resistance cassette (aphAIII),

was amplified from the genomic DNA of the MecA-NTD + GyrA-CTD strain (Qin et al., 2024) using primer pair lucF/R. These three amplicons were assembled via overlap extension PCR (OE-PCR) using primer pair 12005k3F/R. The final OE-PCR product was transformed into the DacA-luciN strain, and kanamycin-resistant colonies were selected to generate the DacA-luciN+luciC strain.

Construction of DacA-luciN+GlmM-luciC strain

A partial open reading frame (ORF) of *glmM* was amplified as 5' flanking region using primer pair 1426luc5fF/R, and its downstream sequence was amplified as 3' flanking region using primer pair 1426luc3fF/R. A third PCR fragment, containing a flexible 3× GGGGS linker followed by the luciferase C-terminal domain (amino acids 156–314) and a kanamycin resistance cassette (aphAIII), was amplified from the genomic DNA of strain MecA-NTD + GyrA-CTD (Qin et al., 2024) using primer pair 1426lucF/R. These three fragments were assembled via overlap extension PCR (OE-PCR) using primer pair 1426luc5k3F/R. The final OE-PCR product was transformed into the DacA-luciN strain, and kanamycin-resistant colonies were selected to generate the DacA-luciN+GlmM-luciC strain.

Construction of DacA-luciN+FruI-luciC strain

A partial open reading frame (ORF) of *fruI* was amplified as 5' flanking region using primer pair 872luc5fF/R, and its downstream sequence was amplified as 3' flanking region using primer pair 872luc3fF/R. A third PCR fragment, containing a flexible 3× GGGGS linker followed by the luciferase C-terminal domain (amino acids 156–314) and a kanamycin resistance cassette (aphAIII), was amplified from the genomic DNA of strain MecA-NTD

+ GyrA-CTD (Qin et al., 2024) using primer pair 872lucF/R. These three fragments were assembled via overlap extension PCR (OE-PCR) using primer pair 872luc5k3F/R. The final OE-PCR product was transformed into the DacA-luciN strain, and kanamycin-resistant colonies were selected to generate the DacA-luciN+FruI-luciC strain.

Construction of DacA-luciN+Ftf-luciC strain

A partial open reading frame (ORF) of *ftf* was amplified as 5' flanking region using primer pair 2028luc5fF/R, and its downstream sequence was amplified as 3' flanking region using primer pair 2028luc3fF/R. A third PCR fragment, containing a flexible 3× GGGGS linker followed by the luciferase C-terminal domain (amino acids 156–314) and a kanamycin resistance cassette (aphAIII), was amplified from the genomic DNA of strain MecA-NTD + GyrA-CTD (Qin et al., 2024) using primer pair 2028lucF/R. These three fragments were assembled via overlap extension PCR (OE-PCR) using primer pair 2028luc5k3F/R. The final OE-PCR product was transformed into the DacA-luciN strain, and kanamycin-resistant colonies were selected to generate the DacA-luciN+Ftf-luciC strain.

Construction of DacA-luciN+SecA-luciC strain

A partial open reading frame (ORF) of *secA* was amplified as 5' flanking region using primer pair 1838luc5fF/R, and its downstream sequence was amplified as 3' flanking region using primer pair 1838luc3fF/R. A third PCR fragment, containing a flexible 3× GGGGS linker followed by the luciferase C-terminal domain (amino acids 156–314) and a kanamycin resistance cassette (aphAIII), was amplified from the genomic DNA of strain MecA-NTD + GyrA-CTD (Qin et al., 2024) using primer pair 1838lucF/R. These three

fragments were assembled via overlap extension PCR (OE-PCR) using primer pair 1838luc5k3F/R. The final OE-PCR product was transformed into the DacA-luciN strain, and kanamycin-resistant colonies were selected to generate the DacA-luciN+SecA-luciC strain.

Construction of DacA-luciN+GapC-luciC strain

A partial open reading frame (ORF) of *gapC* was amplified as 5' flanking region using primer pair 360luc5fF/R, and its downstream sequence was amplified as 3' flanking region using primer pair 360luc3fF/R. A third PCR fragment, containing a flexible 3× GGGGS linker followed by the luciferase C-terminal domain (amino acids 156–314) and a kanamycin resistance cassette (aphAIII), was amplified from the genomic DNA of strain MecA-NTD + GyrA-CTD (Qin et al., 2024) using primer pair 360lucF/R. These three fragments were assembled via overlap extension PCR (OE-PCR) using primer pair 360luc5k3F/R. The final OE-PCR product was transformed into the DacA-luciN strain, and kanamycin-resistant colonies were selected to generate the DacA-luciN+GapC-luciC strain.

Quantification of intracellular c-di-AMP

Intracellular c-di-AMP levels were quantified using a commercially available c-di-AMP ELISA kit (Cayman Chemical) according to the manufacturer's instructions. Briefly, bacterial strains were grown to the log phase, harvested by centrifugation, and washed with phosphate-buffered saline. Cell pellets were lysed by beads beater and clarified lysates were diluted and applied to the ELISA plate. C-di-AMP concentrations were determined by comparison to a standard curve generated using provided c-di-AMP

standards. Measurements were performed using three independent biological replicates, each with three technical replicates. Absorbance was measured at OD_{450} using a Cytation 5 Cell Imaging Multimode Reader, and c-di-AMP levels were calculated and reported as mean ± SD.

Table S1. Strains and plasmids used in this study.

**Strains**	**Description**	**Reference**
UA159	WT *Streptococcus mutans*	(Ajdic et al., 2002)
DacA-FLAG	UA159::□(*dacA*-FLAG)	This study
Δ*dacA*	UA159 Δ*dacA*	(Peng et al., 2016)
Δ502	UA159 Δ502, Erm^{r}	This study
Δ723	UA159 Δ723, Erm^{r}	This study
Δ*fruI*	UA159 Δ*fruI*, Erm^{r}	This study
Δ*ftf*	UA159 Δ*ftf*, Erm^{r}	This study
Δ*dacA*/p*dacA*	UA159 Δ*dacA*/p*dacA*, Spec^{r}	This study
Δ723/p723	UA159 Δ723/p723, Erm^{r}, Spec^{r}	This study
DacA-luciN	UA159::□(*dacA*-luciN), Erm^{r}	This study
723-luciC	UA159::□(723-luciC), Kan^{r}	This study
DacA-luciN+ 723-luciC	UA159::□(*dacA*-luciN , 723-luciC), Erm^{r}, Kan^{r}	This study
DacA3A-luciN+723-luciC	UA159::□(*dacA*3A-luciN , 723-luciC), Erm^{r}, Kan^{r}	This study
DacA-luciN+luciC	UA159::□(*dacA*-luciN , luciC), Erm^{r}, Kan^{r}	This study
DacA-luciN+GlmM-luciC	UA159::□(*dacA*-luciN , *glmM*-luciC), Erm^{r}, Kan^{r}	This study
DacA-luciN+FruI-luciC	UA159::□(*dacA*-luciN , *fruI*-luciC), Erm^{r}, Kan^{r}	This study
DacA-luciN+Ftf-luciC	UA159::□(*dacA*-luciN , *ftf*-luciC), Erm^{r}, Kan^{r}	This study
DacA-luciN+SecA-luciC	UA159::□(*dacA*-luciN , *secA*-luciC), Erm^{r}, Kan^{r}	This study
DacA-luciN+GapC-luciC	UA159::□(*dacA*-luciN , *gapC*-luciC), Erm^{r}, Kan^{r}	This study
**Plasmid**	Description	Reference
pDL278	*E.coli-Streptococcus* shuttle vector, Spec^{r}	(Chen & LeBlanc, 1992)
p*dacA*	pVA380*::dacA,* Spec^{r}	This study

p723	pVA380*::723,* Spec^r	This study

Spec^r, spectinomycin resistance; Erm^r, erythromycin resistance; Kan^r, kanamycin resistance.

Table S2. Primers used in this study.

Primer	Primer sequence (5′-3′)*
14285fF	GAATAAAGCTGGCAATAGCAGA
14285fR	**TTGTAATCGGATCCCCCACCGCC**AACATGATGGCGTCCTCCAAAG
14285fR2	**CTTAAAGTTACTTATCGTCATCGTCC**TTGTAATCGGATCCCCCACCG
14283fF	**ACGATAAGTAACTTTAAGAAGGAGATATACAT**ATGTTTAAGCGGTTTTTACTAAACGTC
14283fR	CTAAAGAATCTTCAACTAACTGAGTC
14283fF2	**GATTACAAGGACGATG**ACGATAAGTAACTTTAAGAAGGAGATATACATATGT
14285fe3F	GCGTTTGAGATACAGAAAAATCACTTG
14285fe3R	GATGCTAAGCATCTTCCTCCA
7235fF	AACAGGGTTTTGGATCGGTTATG
7235fR	**GTTTTGAGAATATTTTATATTTTTGTTCAT**AATAAATTCTCCTTCTCGGTCTTTAAAAG
7233fF	**TATCTATTATTTAACGGGAGGAAATAA**TAGAAAAAATGACTGGTATTGAAACTCC
7233fR	GATGATCTGACAATTACTCAGGAGTC
ErmF	ATGAACAAAAATATAAAATATTCTCAAAAC
ErmR	TTATTTCCTCCCGTTAAATAATAGATAAC
7235e3F	GGTACTTTTGCGACGACGACA
7235e3R	CAAGCCAAAGACTTATTGTAGCCA
5025fF	GTTACTCTTCGTGCAGAGGATG
5025fR	**GTTTTGAGAATATTTTATATTTTTGTTCAT**AATTTCATCCTAATCAGTTCTAAAATAATT
5023fF	**TATCTATTATTTAACGGGAGGAAATAA**AAAATACTTTAATGGATTTTTCAATGAATCC
5023fR	TGTAAACGAACTTGTTTCATTTAATGG
5025e3F	GTTGATAAGATTGAACGTCAGATTCG
5025e3R	GTCACTTATTTGATAGATTAATTCACGATC
8725fF	CAATACGATAATTATTGGTGGTTATGTC
8725fR	**GTTTTGAGAATATTTTATATTTTTGTTCAT**AATTTTTCTACCTCTACTTTTTCATATGTT
8723fF	**TATCTATTATTTAACGGGAGGAAATAA**TTTTCGTTTAATAACAAGTCATATTGTTAGA
8723fR	TCTTAGTTGTTACAGGCACATGC
8725e3F	GTCGGTATGAATGGTATTGATCATC

8725e3R	CTCAATCGTCTCTAACAATGCTGAC
20285fF	AGCCTCTACTTAAAAAACGAGCTG
20285fR	**GTTTTGAGAATATTTTATATTTTTGTTCAT**TAGCAAACCTCCTTTTTTATTATTGTTAC
20283fF	**TATCTATTATTTAACGGGAGGAAATAA**AGCATTTCTTTATATATACAAAACAAAAGAC
20283fR	AGACGTTTGTGAGTACGCATTTC
20285e3F	ACACAGGCTGACAGTAAAAAGTAC
20285e3R	GTAAGCTTTCAAACGGATACGGAT
pva380F	AGAAGATCGATTTTCGTTCGTG
pva380R	CAGATACGGTAAACTAGCCTCGT
1428pF	**ACGAGGCTAGTTTACCGTATCTG**ATGAACATTTTATCATAAAAAAAGCGTTTGAG
1428pR	**CACGAACGAAAATCGATCTTCT**TTAAACATGATGGCGTCCTCCAAAG
723pF	**ACGAGGCTAGTTTACCGTATCTG**TGAAGATTATCAAGGGAGTAGCTAG
723pR	**CACGAACGAAAATCGATCTTCT**TTAAATCGCTTTTTGATCTTGACCAA
1428lucF	**TTCTTTGGAGGACGCCATCATGTT**GGAGGTGGAGGTTCTGGTG
1428lucR	**TGTTCATGTAATCACTCCTTCTTAATTACAAAT**TTAACCCATCCAAGATTCAATAACATC
ErmF2	ATTTGTAATTAAGAAGGAGTGATTACATGAACA
ErmR2	**CTTAAACATATGTATATCTCCTTCTTAAAG**TTATTTCCTCCCGTTAAATAATAGATAACT
1428luc3fF	**CTTTAAGAAGGAGATATACAT**ATGTTTAAGCGGTTTTTTACTAAACGTCTC
1428luc3fR	CAAGAGCTGATTCTAACAATTCTCCAG
1428luc5fF	CACCGTAACGATCCATTTGATC
1428luc5fR	AACATGATGGCGTCCTCCAAAG
1428luc5e3F	CGCACTTAAGAGCAATTTTTCCAGG
1428luc5e3R	ACTATTTGATGCTAAGCATCTTCCTCC
14283Aluc5fF	CCAGCTGGTATCAATGCCATCA
14283Aluc5fR	CTCTTCTATTGATACCAAAGCTCCA
14283Aluc5fF2	TCCTGTCCGTCAATGATGAACTC
14283Aluc5fR2	**GCAATATATTCTCTTAGGGCTGCAGC**CTCTTCTATTGATACCAAAGCTCCA
14283Aluc3fF	GCTGCAGCCCTAAGAGAATATATTGCCACTGGAATTCC
14283Aluc3fR	CAAGAGCTGATTCTAACAATTCTCCAG
14283Aluc5e3F	CAC CGT AAC GAT CCA TTT GAT C
14283Aluc5e3R	ACTATTTGATGCTAAGCATCTTCCTCC
14283Aluc5fF	CCAGCTGGTATCAATGCCATCA
14283Aluc5fR	ATGACTTTAAAATCAAGAATAGGAATTTTAGC
1426lucF	**GTTGTCAAAACAGAAATTGGCATT**GGAGGTGGAGGTTCTGGTG
1426lucR	**GTCTCAGTCCTATACACACTATTT**CTAAAACAATTCATCCAGTAAAATATAATAT
1426luc5fF	CTGGAGAATTGTTAGAATCAGCTCTTG
1426luc5fR	AATGCCAATTTCTGTTTTGACAAC

1426luc3fF	AAATAGTGTGTATAGGACTGAGACAGCTTCTATGATTAT
1426luc3fR	GATCTGGTTCATGAACTAAGATCC
1426luc5k3F	GTCATGATATCTGCTAGTCACAATCC
1426luc5k3R	GACAATAGAATGAATTTCATCTATGAAAAG
723lucF	**GTACTTGGTCAAGATCAAAAAGCGATT**GGAGGTGGAGGTTCTGGTG
723lucR	**GGAGTTTCAATACCAGTCATTTTTTCTA**CTAAAACAATTCATCCAGTAAAATATAAT
723luc5fF	TTCGTCCAATCACTGAGGACGAT
723luc5fR	AATCGCTTTTTGATCTTGACCAAGTAC
723luc3fF	TAGAAAAAATGACTGGTATTGAAACTCCAGTCATT
723luc3fR	TTCCCATTCCATGACGATTTTTGAC
723luc5k3F	AGAAACGGCAGTCGTTGAAAATG
723luc5k3R	CAAATCCTTAACAGTCCATTGAGCT
lucF	**TTTCTTGTCGGAGGTTTTTTTAGA**ATGGGAGGTGGAGGTTCTGGTG
lucR	**TTCAATTTCTACCCATTGATTATAGA**CTAAAACAATTCATCCAGTAAAATATAATAT
12005fF	GTTGTGACTGCAGAAGTCTTAACT
12005fR	TCTAAAAAAACCTCCGACAAGAAATTATAATTCAATATCACCAAACAAG
12003fF	TCTATAATCAATGGGTAGAAATTGAATTTTATTC
12003fR	ATGAAAGTGAAATTAAGCAAACGTTA
12005k3F	GACCAATGATCGTAATGCTGACAT
12005k3R	TGAAGGATCTTGTACACCATCAC
872lucF	**CTTTTTGGAAGTCTTCGTAAAGTAAGA**GGAGGTGGAGGTTCTGGTG
872lucR	**TCTAACAATATGACTTGTTATTAAACGAAAA**CTAAAACAATTCATCCAGTAAAATATAAT
872luc5fF	CTTGATGGTAAAGCTGAAGCCTATG
872luc5fR	TCTTACTTTACGAAGACTTCCAAAAAG
872luc3fF	TTTTCGTTTAATAACAAGTCATATTGTTAGA
872luc3fR	TCTTAGTTGTTACAGGCACATGC
872luc5k3F	TGAAAAAGCTGGTCTTGGAAGT
872luc5k3R	CTCAATCGTCTCTAACAATGCTGAC
2028lucF	**TCTGTGTAAGCATTGGTTTTAAA**GGAGGTGGAGGTTCTGGTG
2028lucR	**GTCTTTTGTTTTGTATATATAAAGAAATGCT**CTAAAACAATTCATCCAGTAAAATATAAT
2028luc5fF	CCACATGTCATTGAAGATGAGAA
2028luc5fR	CCTTTAAAACCAATGCTTACACAGA
2028luc3fF	AGCATTTCTTTATATATACAAAACAAAAGAC
2028luc3fR	GTAAGCTTTCAAACGGATACGGAT
2028luc5k3F	CAAGGTGAAGATCAGATTTACAACT
2028luc5k3R	AAGGCTGATTTTTACAGCACCAAATC

1838lucF	GGAGGTGGAGGTTCTGGTG
1838lucR	CTAAAACAATTCATCCAGTAAAATATAATAT
1838luc5fF	TGCTAGTTCAAGCAGGTGTTC
1838luc5fR	**CACCAGAACCTCCACCTCC**AAATCGCTTGCGTCCATGGCA
1838luc3fF	**ATATTATATTTTACTGGATGAATTGTTTTAG**TGAATTAGAAGGATAAAAAATGTCTT
1838luc3fR	GTATTTGGTTGATGAATGAGGCCT
1838luc5k3F	AGAAGCTCAGATTATTATGAATG
1838luc5k3R	ATTTCCTAAAGTATCATAAATTTCA
360lucF	GGAGGTGGAGGTTCTGGTG
360lucR	CTAAAACAATTCATCCAGTAAAATATAATAT
360luc5fF	TGTTGAAAGGTTGGTTTATATTTGTG
360luc5fR	**CACCAGAACCTCCACCTCC**TTTAGCGATTTTTGCAAAGTACTCAAG
360luc3fF	**ATATTATATTTTACTGGATGAATTGTTTTAG**TACTCATTATTTTGTAAAAGGGGACCTT
360luc3fR	GTATAAGTCATACCACCACCAATAAG
360luc5k3F	ATGGTAGTTAAAGTTGGTATTAACGGT
360luc5k3R	TGAACCACCAAGGATAGCGATA

*Complementary sequences used for overlap extension PCR are shown in bold.

Growth curve measurement

Overnight cultures of *S. mutans* strains were diluted 1:20 in fresh THB and grown to an OD_{600} of 0.4. The cultures were then further diluted 1:100 in fresh THB and incubated in a 96-well plate at 37°C by using Tecan Infinite 200 PRO multimode microplate reader. Cell growth was monitored at OD_{600} every 30 minutes, with a 10-second shaking step before each reading to ensure proper mixing. To investigate the effects of ions on cell growth, chemically defined medium was used in place of THB, and $MnSO_4$ and $MgCl_2$ were added to a final concentration of 100uM and 1mM respectively. The experiment was performed in a triplicate and repeated three times.

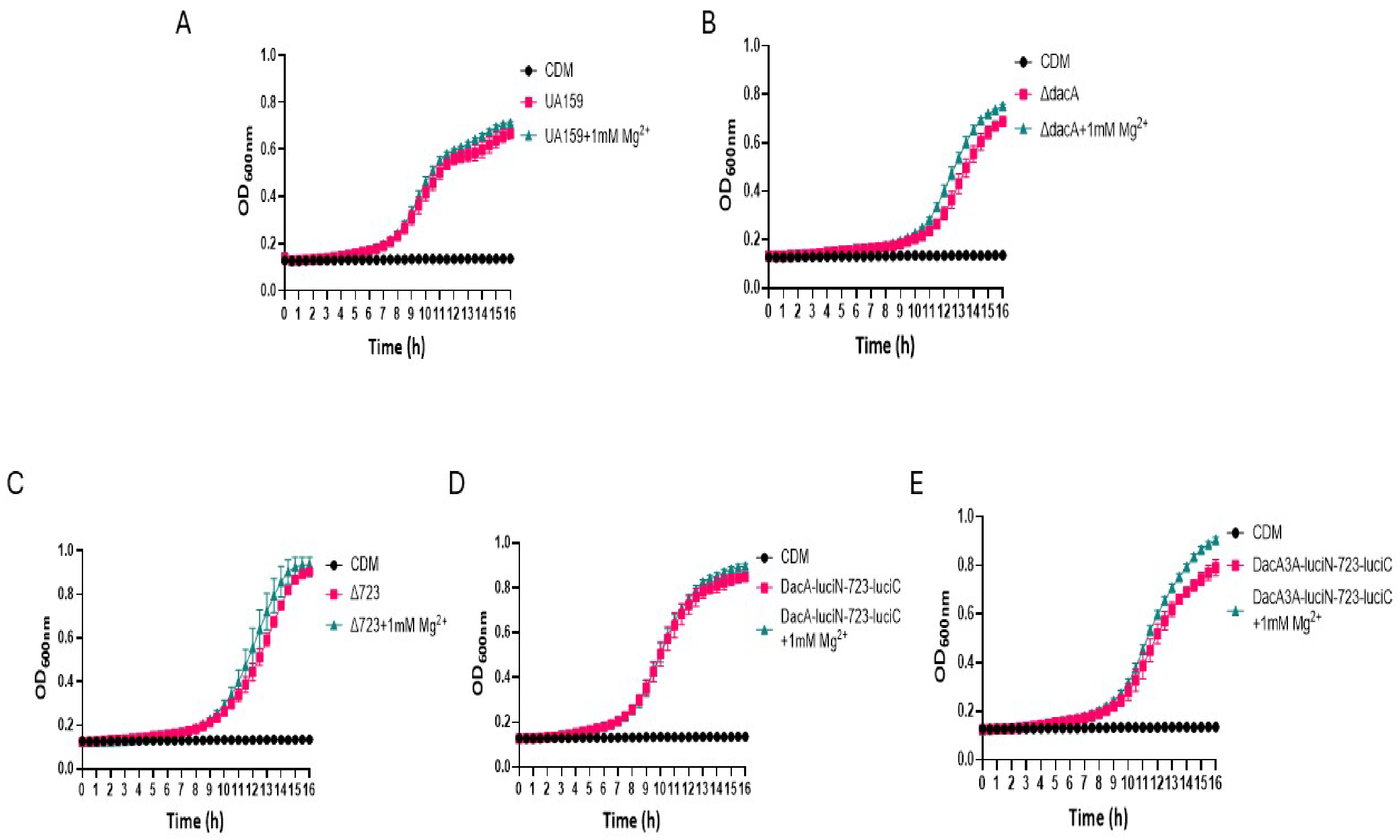
A
B
C
D
E
CDM
UA159
UA159+1mM Mg2+
ΔdacA
ΔdacA+1mM Mg2+
Δ723
Δ723+1mM Mg2+
DacA-luciN-723-luciC
DacA-luciN-723-luciC +1mM Mg2+
DacA3A-luciN-723-luciC
DacA3A-luciN-723-luciC +1mM Mg2+
OD600nm
Time (h)

Fig. 1S. Growth analysis of S. mutans under Mg^{2+} Supplementation. Growth curves of wild-type strain UA159 (A), deletion strain ΔdacA (B), Δ723 strain (C), DacA-luciN+723-luciC strain (D) and site mutated strain DacA3A-luciN+723-luciC (E) were measured in chemical defined media with or without 1 mM magnesium. The figures present a representative result from three independent assays; each performed with three technical replicates. Data are presented as mean ± SD.

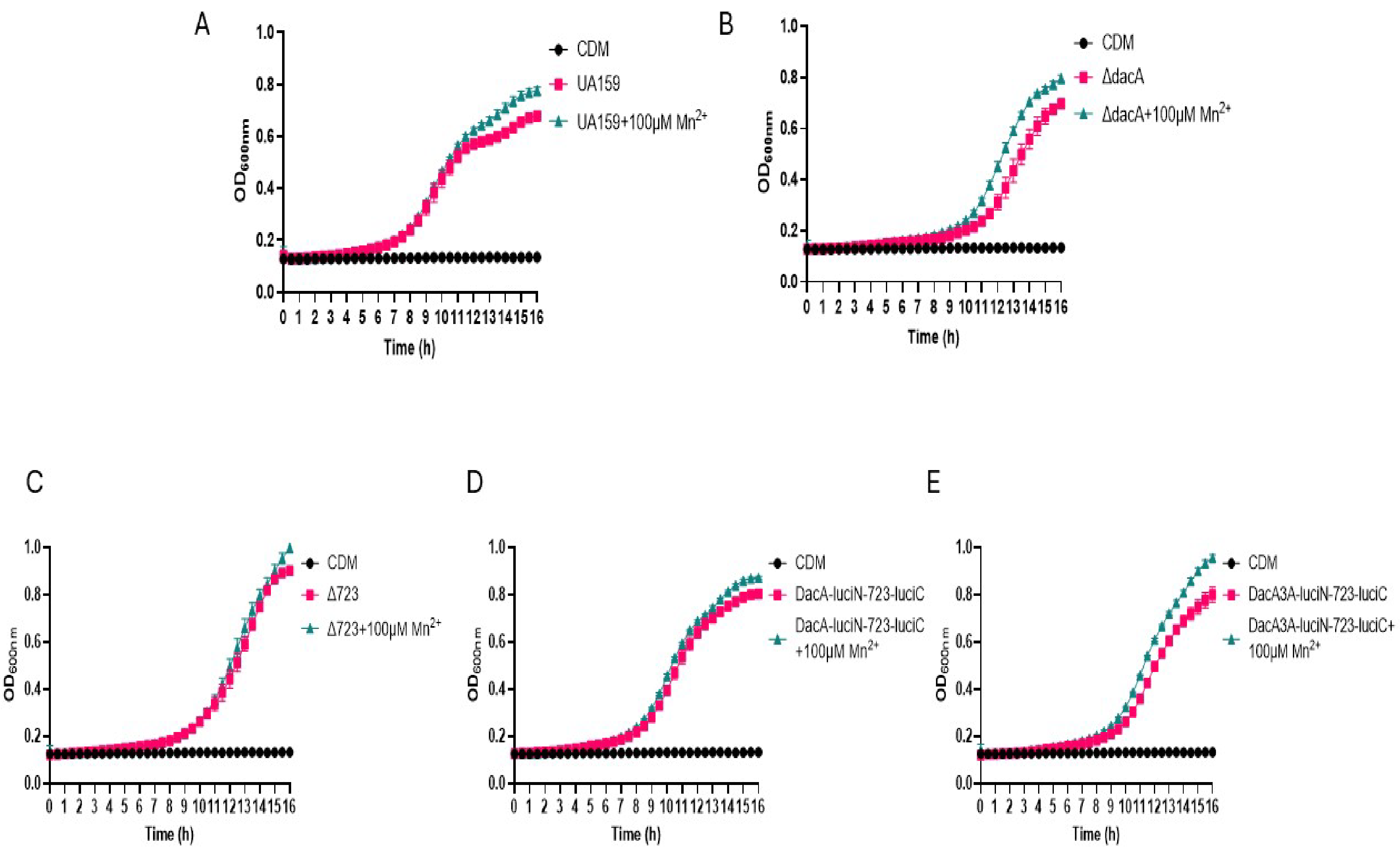
A
1.0
0.8
0.6
0.4
0.2
0.0
OD600nm
0 1 2 3 4 5 6 7 8 9 10 11 12 13 14 15 16
Time (h)
CDM
UA159
UA159+100μM Mn2+
B
OD600nm
Time (h)
CDM
ΔdacA
ΔdacA+100μM Mn2+
C
OD600nm
Time (h)
CDM
Δ723
Δ723+100μM Mn2+
D
OD600nm
Time (h)
CDM
DacA-luciN-723-luciC
DacA-luciN-723-luciC
+100μM Mn2+
E
OD600nm
Time (h)
CDM
DacA3A-luciN-723-luciC
DacA3A-luciN-723-luciC+
100μM Mn2+

Fig. 2S. Growth analysis of S. mutans under Mn^{2+} Supplementation. Growth curves of wild-type strain UA159 (A), deletion strain ΔdacA (B), Δ723 strain (C), DacA-luciN+723-luciC strain (D) and site mutated strain DacA3A-luciN+723-luciC (E) were measured in chemical defined media with or without 100 μM manganese. The figures present a representative result from three independent assays; each performed with three technical replicates. Data are presented as mean ± SD.

Reference

Ajdic, D., McShan, W. M., McLaughlin, R. E., Savic, G., Chang, J., Carson, M. B., Primeaux, C., Tian, R., Kenton, S., Jia, H., Lin, S., Qian, Y., Li, S., Zhu, H., Najar, F., Lai, H., White, J., Roe, B. A., & Ferretti, J. J. (2002). Genome sequence of Streptococcus mutans UA159, a cariogenic dental pathogen. *Proc Natl Acad Sci U S A*, *99*(22), 14434-14439. https://doi.org/10.1073/pnas.172501299

Chen, Y. Y., & LeBlanc, D. J. (1992). Genetic analysis of scrA and scrB from Streptococcus sobrinus 6715. *Infect Immun*, *60*(9), 3739-3746. https://doi.org/10.1128/iai.60.9.3739-3746.1992

Gibson, D. G. (2011). Enzymatic assembly of overlapping DNA fragments. *Methods Enzymol*, *498*, 349-361. https://doi.org/10.1016/B978-0-12-385120-8.00015-2

Peng, X., Michalek, S., & Wu, H. (2016). Effects of diadenylate cyclase deficiency on synthesis of extracellular polysaccharide matrix of Streptococcus mutans revisit. *Environ Microbiol*, *18*(11), 3612-3619. https://doi.org/10.1111/1462-2920.13440

Qin, H., Anderson, D., Zou, Z., Higashi, D., Borland, C., Kreth, J., & Merritt, J. (2024). Mass spectrometry and split luciferase complementation assays reveal the MecA protein interactome of Streptococcus mutans. *Microbiol Spectr*, *12*(2), e0369123. https://doi.org/10.1128/spectrum.03691-23

Qin, H., Zou, Z., Anderson, D., Sang, Y., Higashi, D., Kreth, J., & Merritt, J. (2021). The transcription regulator BrsR serves as a network hub of natural competence protein-protein interactions in Streptococcus mutans. *Proc Natl Acad Sci U S A*, *118*(39). https://doi.org/10.1073/pnas.2106048118
